# HOPS: automated detection and authentication of pathogen DNA in archaeological remains

**DOI:** 10.1186/s13059-019-1903-0

**Published:** 2019-12-16

**Authors:** Ron Hübler, Felix M. Key, Christina Warinner, Kirsten I. Bos, Johannes Krause, Alexander Herbig

**Affiliations:** 1 Max Planck Institute for the Science of Human History, Jena, Germany; 2 Institute for Medical Engineering and Sciences, Massachusetts Institute of Technology, Cambridge, MA 02139 USA; 3 Department of Civil and Environmental Engineering, Massachusetts Institute of Technology, Cambridge, MA 02139 USA

**Keywords:** Ancient DNA, Archaeogenetics, Pathogen detection, Metagenomics, Paleopathology, Ancient bacteria, Microbial archaeology

## Abstract

High-throughput DNA sequencing enables large-scale metagenomic analyses of complex biological systems. Such analyses are not restricted to present-day samples and can also be applied to molecular data from archaeological remains. Investigations of ancient microbes can provide valuable information on past bacterial commensals and pathogens, but their molecular detection remains a challenge. Here, we present HOPS (Heuristic Operations for Pathogen Screening), an automated bacterial screening pipeline for ancient DNA sequences that provides detailed information on species identification and authenticity. HOPS is a versatile tool for high-throughput screening of DNA from archaeological material to identify candidates for genome-level analyses.

## Background

High-throughput DNA sequencing enables large-scale metagenomic analyses of environmental samples and host tissues and provides an unprecedented understanding of life’s microbial diversity. Examples of coordinated efforts to quantify this diversity include the Human Microbiome Project [1], the Tara Ocean Project [2], and the Earth Microbiome Project [3]. Metagenomic data from human archaeological remains (e.g., bones, teeth, or dental calculus) provide a window into the individuals’ metagenomic past and are an unprecedented temporal dimension added to the wide landscape of microbial diversity now being explored. While many ancient DNA (aDNA) studies focus on the analysis of human endogenous DNA isolated from ancient specimens [4–8], co-recovery of metagenomic aDNA permits queries that provide information related to endogenous microbial content at death, with applications ranging from characterizing the natural constituents of the microbiota to identifying infectious diseases [9, 10].

Genome-level investigations of ancient bacterial pathogens have provided valuable information about the evolution of *Yersinia pestis* [11–18], *Mycobacterium leprae* [19, 20], *Mycobacterium tuberculosis* [21, 22], pathogenic *Brucella* species [23, 24], *Salmonella enterica* [25, 26], and *Helicobacter pylori* [27], with others surely on the horizon. Notably, most studies to date have leveraged paleopathological evidence or historical context to pinpoint a priori involvement of a specific bacterial pathogen. However, the vast majority of infectious diseases do not lead to the formation of distinct and characteristic bone lesions, and most remains are found in contexts that lack clear associations with a particular disease. Consequently, studies of ancient pathogens must consider a long list of candidate microbes. Given the sizes and availability of current aDNA datasets, there is clear benefit for the development of an automated computational screening tool that both detects and authenticates true pathogen genetic signals in ancient metagenomic data. Ideally, this tool also is able to distinguish pathogens from the dominant and diverse microbial background of archaeological and other decomposed material, a consideration typically not required for tools developed for clinical applications.

To save computational time and effort, most available metagenomic profiling tools focus only on individual genes, such as the 16S rRNA gene used by QIIME [28], or panels of marker genes, such as those used by MetaPhlAn2 [29] and MIDAS [30], that are easy to retrieve and sufficiently specific. However, these genes make up only a small proportion of a bacterial genome (the 16S rRNA gene, for example, accounts for only ~ 0.2% of a bacterial genome and is usually present in multiple copies), and if a pathogen is present at low abundance compared to host and environmental DNA, these genes are likely to be missed in routine metagenomic sequencing screens. Although these tools can have high specificity, they lack the sensitivity required for ancient pathogen screening from shallow but highly complex metagenomic datasets. Screening techniques that accommodate queries of whole genomes are of clear benefit for archaeological studies since alignment to a full reference genome offers greater chances for detection when data for a given taxon are sparse [25]. While some algorithms, such as Kraken [31], have been developed to query databases that contain thousands of complete reference genomes using k-mer matching, this approach does not produce the alignment information necessary to further evaluate species identification accuracy or authenticity.

In addition to taxonomic classification [32], it is also helpful to distinguish ancient bacteria from modern contaminants as early as the initial screening [9, 10]. Genuine aDNA, especially pathogen bacterial DNA, is usually only present in small amounts and can be distinguished from modern DNA contamination by applying an established set of authenticity criteria [9, 10], the most important of which is the assessment of DNA damage. In ancient DNA, cytosine deamination accumulates over time at DNA fragment termini [9, 10, 33, 34], thus leading to a specific pattern of nucleotide misincorporation during amplification. The evaluation of additional authenticity criteria such as edit distances (number of mismatches between read and reference) and the distribution of mapped reads across the reference are also recommended to circumvent database bias artifacts and to further validate taxonomic assignments [9, 10]. While manual evaluation of species identification and aDNA authenticity using standalone tools might be feasible for a small sample set, it is impractical for the large sample sizes typical of recent ancient DNA investigations. The increasing throughput of the ancient DNA field warrants an automated high-throughput solution for pathogen detection in metagenomic datasets.

Successful ancient pathogen detection is reliant upon three criteria: (i) specificity of species-level detection against a diverse metagenomic background, (ii) high sensitivity that allows detection even with a weak signal when only trace amounts of species-specific DNA are present, and (iii) authentication of its ancient origin. No software currently exists that fulfills all requirements for reliable screening of metagenomic aDNA. Here, we introduce HOPS (Heuristic Operations for Pathogen Screening), an automated computational pipeline that screens metagenomic aDNA data for the presence of bacterial pathogens and assesses their authenticity using established criteria. We test HOPS on experimental and simulated data and compare it to common metagenomic profiling tools. We show that HOPS outperforms available tools, is highly specific and sensitive, and can perform taxonomic identification and authentication with as few as 50 species-derived reads present.

## Results

### HOPS workflow

**Fig. 1:**
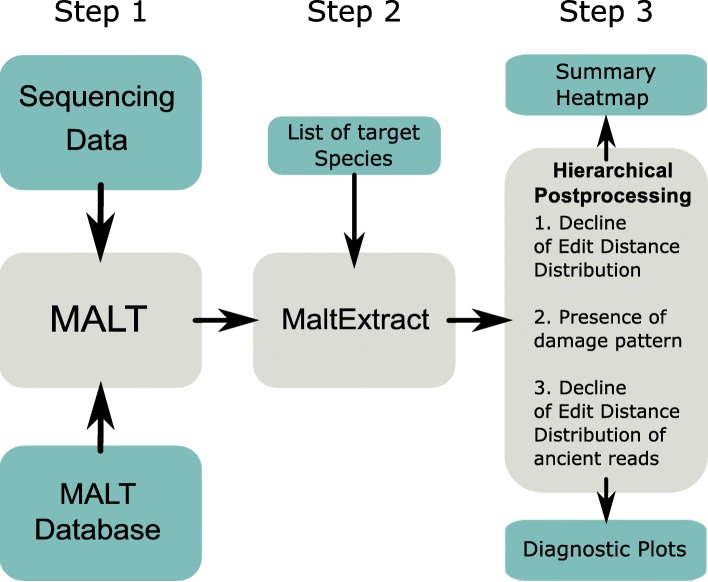
Schematic depiction of HOPS workflow. First, MALT aligns the metagenomic data against its reference database and has an optional mode for processing aDNA reads. MaltExtract then processes the MALT output with various filters and produces various statistics. Finally, post-processing procedures provide a comprehensive visualization of the output that can be evaluated to identify putatively positive hits

HOPS consists of three parts (Fig. 1): (i) a modified version of MALT [25, 35] that includes optional PCR duplicate removal and optional deamination pattern tolerance at the ends of reads; (ii) the newly developed program MaltExtract that provides statistics for the evaluation of species identification as well as aDNA authenticity criteria for an arbitrarily extensive user-specified set of bacterial pathogens, with additional functionality to filter the aligned reads by various measures such as read length, sequence complexity, or percent identity; and (iii) a post-processing script that provides a summary overview for all samples and potential bacterial pathogens that have been identified.

### MALT

MALT (Megan Alignment Tool) [25, 35] is an alignment and taxonomic binning tool for metagenomic data that aligns DNA reads to a user-specified database of reference sequences. Reads are assigned to taxonomic nodes by the naïve Lowest Common Ancestor (LCA) algorithm [36, 37] and are thus assigned to different taxonomic ranks based on their specificity. The default version of MALT is intended for the analysis of metagenomic datasets derived from modern DNA, and thus, it was not designed to accommodate the specific requirements of aDNA analyses. In particular, aDNA damage that manifests as misincorporated nucleotides in sequenced products can lead to an increased number of mismatches, and extensive damage has the potential to prevent alignment or alter taxonomic assignment. Loss of target reads due to DNA damage can hamper species detection since aDNA studies usually begin with shallow sequence data for initial evaluations of sample quality. In addition, archaeological remains often have low DNA yields, and library amplification can result in a high number of PCR duplicates that can falsely inflate quantitative estimates of taxa.

To accommodate such established phenomena, we introduce a new version of MALT that is specifically tailored to the analysis of aDNA data. In this modified version, PCR duplicates are removed by eliminating reads identical to those already aligned. In addition, reads are optionally filtered for a minimum Wootton and Federhen complexity [38] in order to remove reads with low sequence complexity. Furthermore, C>T substitutions are ignored in the first five positions from the 5′-end and G>A in first five positions from the 3′-end, thus removing the influence of aDNA damage on alignment scores.

### HOPS’ authentication strategy

The core of HOPS is formed by the newly developed MaltExtract module. Without MaltExtract, the result files produced by MALT (RMA6 format) can only be evaluated manually with the metagenomic analysis tool MEGAN [39]. Such analysis becomes infeasible when working with large data sets, wherein each sample must be separately searched for a long list of candidate organisms, a process that is both laborious and prone to subjectivity. MaltExtract provides an automated approach for the assessment of the alignment information stored in RMA files generated by MALT. It automatically retrieves and assesses information on various evaluation criteria for all taxonomic nodes that match a given list of target species.

MaltExtract obtains information on edit distance distribution, read length distribution, coverage distribution and alignment mismatch patterns in order to identify and authenticate the presence of species-specific aDNA. Furthermore, MaltExtract allows data filtering for maximum read length, minimum percent identity, minimum complexity, and aDNA damage pattern.

**Fig. 2:**
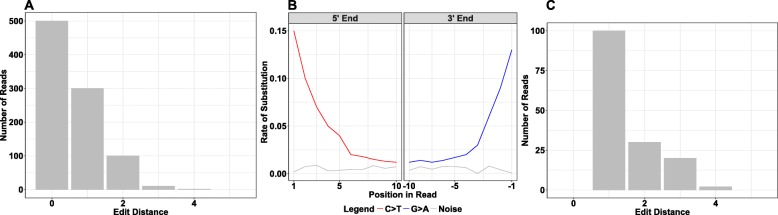
Post-processing steps in HOPS. Three hierarchical post-processing steps are used in HOPS. **a** First, the edit distance distribution is required to show a decline. **b** Second, the alignments are assessed for C>T and G>A mismatches typical for aDNA; by default, any such damage is considered sufficient. **c** Third, the edit distance distribution of reads showing damage is evaluated

Accuracy in taxonomic read assignment is evaluated in a three-step procedure that includes ancient authentication criteria (Fig. 2). The first step evaluates the read assignment to a taxonomic node. Incorrect read assignments can occur when databases are incomplete: many species in a metagenomic sample may have no representative reference genome in the database, and hence their individual reads may become erroneously assigned to the taxon showing the closest genetic match, which could belong to a different species or genus. Mapping to an incorrect species generally results in an increased number of mismatches across the read that is evident in the edit distance distribution (Fig. 2a). By contrast, if the sequenced reads are assigned to the correct reference species, the edit distance distribution should continuously decline, with most of the reads showing no or only a few mismatches that mostly resulted from aDNA damage or evolutionary divergence of the modern reference from the ancient genome. We summarize the shape of the edit distance distribution by a score we term the *negative difference proportion (−Δ%)*, which leverages the difference in sequencing read counts between neighboring mismatch categories (Additional file 1: Figure S1). The − Δ% takes values between 0 and 1, where 1 indicates a strictly declining edit distance distribution. While true positives have a − Δ% of 1 when enough endogenous species-specific sequencing reads are present, we use a threshold of − Δ% > 0.9 to account for possible perturbations due to stochasticity in the edit distance distribution when few reads (~ 10–20) are present. As such, this permits the detection of very low abundant taxa.

In a second step, the ancient origin of the DNA is evaluated through analysis of DNA miscoding lesion patterns (Fig. 2b). The most prominent modification observed is deamination of cytosine into uracil, which is read as a thymine by the polymerase. This leads to an overrepresentation of C>T substitutions at the 5′ end and correspondingly G>A substitutions at the 3′ end [9, 10, 34, 40]. Evaluation of damage patterns is mandatory in any ancient DNA study. MaltExtract reports the rates of substitutions for the leading and trailing 10 positions of the read alignment. The default post-processing settings require only a single miscoding lesion to be present in at least one read for the assigned taxon to qualify as exhibiting damage. This maximizes sensitivity and allows authentication to function largely independently of read depth.

As a third and final criterion, we evaluate the accuracy of taxonomic assignment for all aligned reads exhibiting aDNA damage. For this, we assess again the edit distance distribution using the − Δ% score, but now this is only performed for damaged reads (Fig. 2c) and thus all reads harbor by definition at least one mismatch. In this step, a greater number of assigned reads (> 100) is required for reliable edit distance evaluation due to the fact that not all ancient reads are expected to exhibit damage.

The MaltExtract output is saved in a structured output folder with a summary file of the processed input and subfolders for each evaluation criterion. The post-processing tool generates a summary highlighting which of the target species passed one or more evaluation criteria for each sample, as well as detailed diagnostic plots displaying the evaluation criteria for each supported target species (Additional file 1: Figure S2). Using the versatile MaltExtract output additional post-processing scripts can be developed to extract user-defined criteria, as for instance the GUI-based MEx-IPA (https://github.com/jfy133/MEx-IPA).

### Assessment of taxonomic assignment on simulated data

The naïve LCA algorithm [36], which is part of HOPS, assigns reads to different taxonomic levels depending on the specificity of sequence matches. Taxonomic assignment thus depends on the structure of the underlying reference database, and it is critical to understand the expected taxonomic placement of sequenced reads from each microbial pathogen in order to successfully identify them.

To analyze the taxonomic placement of a test set of 33 bacterial pathogens and to assess the performance of HOPS, we simulated sequencing reads that included artificial DNA damage and spiked them into dentine, dental calculus, bone, and soil metagenomic backgrounds (see Table 1).

**Table 1:** Metagenomic backgrounds used for simulated data sets

ID	Source	Age (Period)	Treatment	Reference
KT31calc	Calculus	Medieval	No UDG	[41]
LP39.10	Dentine	2920–2340 BCE	No UDG	[42]
MK5.001	Dentine	3348–3035 BCE 3619–3366 BCE	UDG half	[43]
TÖSM_1a	Bone	6000–5500 BCE	UDG half	[44]
Soil	Soil	–	No UDG	[25]

**Fig. 3:**
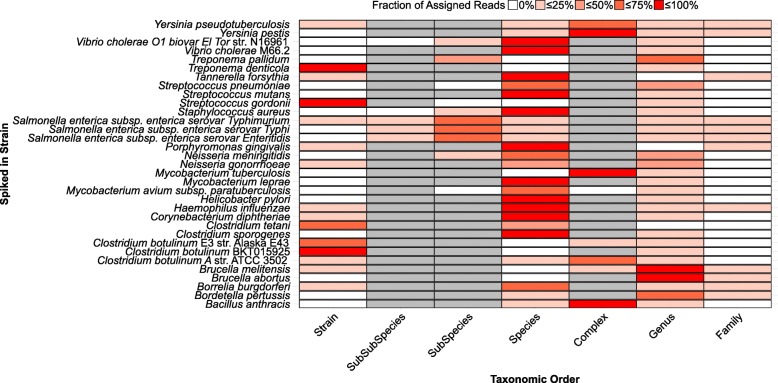
Assignment of simulated reads to taxonomic levels for 33 bacterial pathogens. The fraction of simulated reads (red gradient) per reference (*y*-axis) assigned to a specific node across different levels of the taxonomy (*x*-axis). The levels of taxonomy not defined for a species are shown in gray

Applying the HOPS pipeline, we recovered 98% of the simulated reads for 32 of the 33 bacterial taxa of interest (Fig. 3). The one exception was *Mycobacterium avium subsp. paratuberculosis* K10 for which 23% of simulated reads were assigned to a different *Mycobacterium avium subsp. paratuberculosis* strain. Our analysis shows that in most cases the vast majority of the simulated pathogen reads are assigned to the taxonomic levels “species” and “complex” (e.g., *Mycobacterium tuberculosis* complex and *Yersinia pseudotuberculosis* complex). Noteworthy exceptions were *Brucella abortus*, *Brucella melitenis*, and *Bordetella pertussis*. Upon further investigation, we found that many species within the genera *Brucella* and *Bordetella* show a high degree of sequence similarity, thus causing the majority of the reads deriving from these pathogens to be assigned at the genus level. By contrast, read assignment was found to be very specific for five taxa (*Treponema denticola* ATCC 35405, *Clostridium tetani* E89, *Clostridium botulinum* E3 str. Alaska E43*, Streptococcus gordonii* str. Challis substr. CH1 and *Clostridium botulinum* BKT015925), resulting in the majority of reads deriving from these taxa to be assigned at the strain level. For *Salmonella enterica subsp. enterica*, most reads were assigned at the subspecies level. The results of this test provide a guide for the levels of taxonomic identification that should be considered when searching for any of the 33 queried bacterial species in experimental ancient datasets. Further, it provides a framework to assess taxonomic placement and subsequent identification for other ancient microbes.

### Optimization of MALT for aDNA

**Fig. 4:**
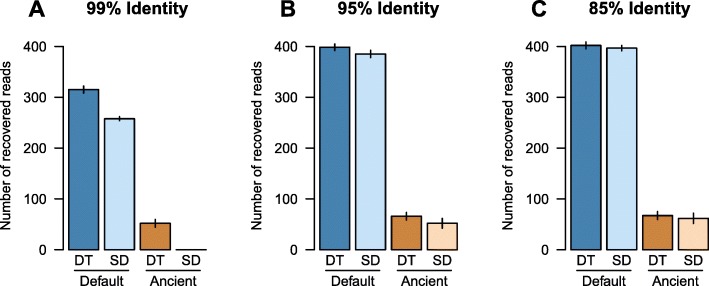
Comparison of the number of successfully recovered *Y. pestis* reads using standard (SD) and damage-tolerant (DT) MALT with minimum percent identities of **a** 99%, **b** 95%, and **c** 85%. Shown are the recovered reads from the “default” (all reads) and “ancient” (reads with damage) modes in MALT, with the same 500 reads being spiked into the metagenomic backgrounds. Error bars show the standard error of five independent technical replicates for each analysis

Because MALT was designed for taxonomic binning of modern genetic data, adapting it to be used on aDNA required altering the original MALT implementation to tolerate terminal substitutions consistent with aDNA damage so that they would not interfere with the percent identity filter. To evaluate the efficacy of this modification, we compared the performance of the modified, damage tolerant version of MALT to the default version using simulated *Y. pestis* data with high terminal damage (~ 40%) and three different percent identity filters: 85%, 95%, and 99% (Fig. 4).

As expected, the greatest difference was observed when applying the stringent 99% identity filter, for which the damage tolerant MALT version recovered ~ 20% more reads than the standard MALT version. Additionally, only the modified version was able to recover reads with simulated damage under these parameters. At 95% identity, only a small difference could be observed between the two MALT versions, while results were almost identical at an 85% identity level. Taken together, the damage tolerant MALT version provides an advantage when searching for a given pathogen using stringent filtering criteria.

### Performance comparison of HOPS, Kraken, SPARSE, MIDAS, and metaBIT on simulated data

We evaluated the performance of HOPS by comparing it to four metagenomic profiling tools: MIDAS [30], a marker gene-based taxonomic classifier, Kraken [31], which performs taxonomic classification based on k-mer matching to a database of complete genomes, metaBIT [45], a pipeline designed for the assessment of ancient metagenomes, and SPARSE [46], which uses a reduced, structured database and a probabilistic model for accurate species assignment. The marker gene database of MIDAS lacked representation for *Yersinia pseudotuberculosis*, *Bordetella pertussis*, and *Brucella melitensis.* Therefore, MIDAS could only be evaluated for 30 of the 33 bacterial pathogens in the simulated data sets. For Kraken, we downloaded the bacterial database, which lacked a reference genome to *Clostridium sporogenes*.

**Fig. 5:**
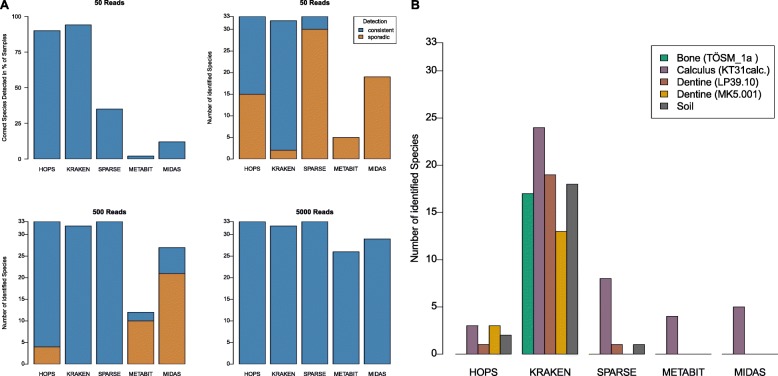
Performance comparison of HOPS, Kraken, SPARSE, metaBIT, and MIDAS. **a** Number of species that have been correctly identified in the simulated data sets by each of the programs. The bar plot on the upper left shows the percentage of data sets with 50 simulated reads for which the correct species has been identified. The other bar plots show the number of species that have been correctly identified in data sets with 50, 500, and 5000 simulated reads, respectively. **b** Number of target species identified in the metagenomic background (negative controls) without any spiked-in species-derived data for each of the tested programs

HOPS consistently detected all 33 pathogens in all backgrounds and among replicates with as few as 50 reads (see Fig. 5a). However, for 15 species, authentication could not be performed in all cases due to the low number of reads. With 50 species-derived reads, HOPS could positively authenticate species assignment in 90% of all tests. For 500 reads, authentication succeeded for all species except for *Streptococcus gordonii*, *S. pneumonia*, *Neisseria gonorrhoeae* and *N. meningitidis*. These species were found in all data sets but authentication was not possible in dental calculus due to a strong background of other *Streptococcus* and *Neisseria* species. Kraken failed to identify *Brucella abortus* and *Mycobacterium tuberculosis* in some replicates with only 50 simulated pathogen reads, resulting in a sensitivity of 94%; however, it was prone to a high false positive rate (see below). SPARSE only sporadically detected species represented by 50 reads (sensitivity of 35%) with only three species consistently identified across all backgrounds (*B. melitensis*, *C. tetani*, and *T. denticola*). However, SPARSE showed a sensitivity of 100% when 500 or 5000 simulated species-derived reads were present. The sensitivity of MIDAS and metaBIT were far lower than for Kraken, SPARSE, and HOPS. Even with 500 simulated pathogen reads, most species were only sporadically detected (i.e., not in all backgrounds/replicates) or were not detected at all. With 5000 simulated reads, however, MIDAS detected 29 of the 30 possible bacterial pathogens. metaBIT, which integrates MetaPhlAn2 [29], detected 26 pathogens under the same conditions. This can be explained by the lower sensitivity of marker gene-based approaches, which require relatively high sequencing coverage in order to ensure adequate representation of the genes needed for identification. This is further evident since MIDAS’ and metaBIT’s sensitivities are correlated with an increase in the number of simulated reads, which has less of an influence for Kraken, SPARSE, and HOPS.

### Negative controls

To assess false positive assignments, we queried all five metagenomic datasets prior to the addition of simulated pathogen reads for detectable signatures of the 33 test bacterial pathogens using Kraken, SPARSE, MIDAS, metaBIT, and HOPS. Kraken showed the highest susceptibility to false positives (see Fig. 5b; Additional file 1: Table S1). Of the 33 pathogens considered, Kraken detected 24 (73%) in calculus, 19 (58%) in dentine, 13 (39%) in bone, and 18 (55%) in soil. Most problematically, *Mycobacterium tuberculosis* and *Bordetella pertussis* were detected by Kraken in every metagenomic background.

SPARSE detected oral streptococci, *Tannerella forsythia*, *Treponema denticola*, and *Porphyromonas gingivalis* as well as *Haemophilus influenzae* and *Neisseria meningitidis* in the calculus background. Furthermore, *Clostridium botulinum* was detected in dentine and *Clostridium tetani* in soil.

MIDAS and metaBIT detected only oral streptococci, *Tannerella forsythia, Treponema denticola*, and *Porphyromonas gingivalis* in the calculus background. Overall, both tools produced fewer identifications than Kraken and SPARSE, but such a result is expected given their reliance on marker gene-based detection, which limits identification to only abundant taxa.

HOPS detected and authenticated four test pathogens in the metagenomic background datasets: *Clostridium tetani* (soil), *Streptococcus mutans* (calculus, dentine), *Treponema denticola* (calculus, dentine), and *Porphyromonas gingivalis* (calculus only)*.* Because *C. tetani* is ubiquitous in soil, and all other detected bacteria are commensals of the human oral cavity, their identification likely reflects true positives. In addition to these four pathogens, there was a weak detection of *Neisseria meningitidis* in dentine. Compared to Kraken, HOPS, SPARSE, MIDAS, and metaBIT all produce only few false positive assignments. Kraken’s increased vulnerability for aberrant assignments likely relates to the absence of an alignment step, which is necessary for reliable species evaluation in both modern and ancient contexts.

### Positive controls

In addition to performing tests using simulated data, we also tested HOPS, Kraken, SPARSE, metaBIT, and MIDAS on 25 ancient metagenomic datasets known to be positive for bacterial pathogens (Table 2). They consisted of both shotgun and capture data and they varied in sequencing depth in accordance with experimental conditions and method of data generation.

**Table 2:** Metagenomic samples used as positive controls

ID	Reconstructed Bacteria	Sequencing reads	Data type	Detected	Reference
10C	*Salmonella enterica*	1,017,400	Shotgun	HP, KA, MB, MI, SP	[25]
35C	*Salmonella enterica*	986,908	Shotgun	HP, KA, MI, SP	[25]
RK1001.C0101	*Yersinia pestis*	7,023,370	Shotgun	HP, KA, MI, SP	[17]
GEN_72	*Yersinia pestis*	7,663,408	Shotgun	HP, KA, MB, SP	[17]
549_O	*Yersinia pestis*	1,520,471	Shotgun	HP, KA, MI, SP	[16]
JK3031UDG	*Yersinia pestis*	4,059,016	Shotgun (UDG)	HP, KA, MI, SP	[16]
JK2370UDG	*Yersinia pestis*	52,858,027	Shotgun (UDG)	HP, KA, MB, MI, SP	[16]
RT6	*Yersinia pestis*	6,706,316	Shotgun (UDG)	HP, KA	[18]
1343UnTal85	*Yersinia pestis*	3,462,216	Shotgun	HP, KA, MB, MI, SP	[17]
6Post	*Yersinia pestis*	2,546,695	Shotgun	HP, KA, MB, MI, SP	[17]
KunilaII	*Yersinia pestis*	1,007,417	Shotgun	HP, KA, MB, MI, SP	[17]
RISE00	*Yersinia pestis*	6,000,000	Shotgun	HP, KA, MI, SP	[13]
RISE139	*Yersinia pestis*	6,000,000	Shotgun	HP, KA, MB, MI, SP	[13]
RISE386	*Yersinia pestis*	6,000,000	Shotgun	HP, KA, MI, SP	[13]
RISE397	*Yersinia pestis*	6,000,000	Shotgun	HP, KA, SP	[13]
RISE505	*Yersinia pestis*	6,000,000	Shotgun	HP, KA, MB, MI, SP	[13]
RISE509	*Yersinia pestis*	6,000,000	Shotgun	HP, KA, MB, MI, SP	[13]
RISE511	*Yersinia pestis*	6,000,000	Shotgun	HP, KA, SP	[13]
54	*Mycobacterium tuberculosis*	70,897	Shotgun	HP, KA, MI, SP	[21]
58	*Mycobacterium tuberculosis*	114,555	Shotgun	HP, KA, MI, SP	[21]
64	*Mycobacterium tuberculosis*	160,310	Shotgun	HP, KA, MB, MI, SP	[21]
54	*Mycobacterium tuberculosis*	5,000,000	Capture (UDG)	HP, KA, MB, MI, SP	[21]
58	*Mycobacterium tuberculosis*	5,000,000	Capture (UDG)	HP, KA, MB, MI, SP	[21]
64	*Mycobacterium tuberculosis*	5,000,000	Capture (UDG)	HP, KA, MB, MI, SP	[21]
P1P2	*Helicobacter pylori*	5,000,000	Capture (UDG)	HP, KA, MB, MI, SP	[27]

*HP* HOPS, *KA* KRAKEN, *MB* metaBIT, *MI* MIDAS, *SP* SPARSE

HOPS and Kraken share 100% sensitivity for the detection of target bacterial pathogens in every sample. SPARSE only failed to detect *Y. pestis* in the sample RT6. By contrast, MIDAS and metaBIT only detected the correct bacterial pathogen in 22 and 14 out of 25 samples, respectively. Again, their sensitivity was likely reduced due to the marker gene-based approach. These results highlight the advantage of whole-genome based approaches like MALT, SPARSE, and Kraken that take advantage of every sequenced read.

### Runtimes

To calculate the runtime for each program, we used five million simulated metagenomic sequencing reads (see “Methods”). For each file, HOPS required an average of 3307 ± 820 s for the MALT step, 16 ± 1 s for the MaltExtract step, and 1 ± 0 s for post processing, for a total of approximately 55 min of analysis time per file. Kraken took on average 72 ± 16 s to run *Kraken_alignment* and 22 ± 3 for *Kraken_translate*, for a total of 1.5 min. The SPARSE analysis took on average 5653 ± 1293 s (about 94 min) for each sample. The MIDAS pipeline processed each file in an average of 73 ± 4 s, and metaBIT needed on average 10 s per sample. HOPS and SPARSE by far required the highest runtimes of the tested tools, but most of this time was required for sequence alignment, a step that, although time consuming, increases detection sensitivity, reduces false positives, and enables the authentication of aDNA reads.

For these tests HOPS, Kraken, SPARSE, MIDAS, and metaBIT were run with 450 GB, 100 GB, 100 GB, 1 GB, and 10 GB of main memory, respectively.

## Discussion

The field of archaeogenetics faces several challenges, such as the low amount of endogenous target DNA, the highly degraded molecules, and unknown and diverse metagenomic backgrounds that accumulate during decomposition and centuries spent in a depositional environment. These factors complicate reliable identification and authentication of genuine ancient DNA, particularly when the targeted bacterial DNA is present in small amounts. Furthermore, many bacterial pathogens have close relatives in soil, which necessitates careful selection of reference sequences as well as meticulous care when making pathogen identifications (see [9, 10] for reviews discussing these challenges).

HOPS provides an automated pipeline for high-throughput ancient bacterial species detection and authentication from metagenomic sequencing data. We compare HOPS to Kraken, SPARSE, metaBIT, and MIDAS, several widely used methods that estimate both the presence and abundance of bacterial taxa in metagenomic data. Aside from metaBIT and SPARSE, these tools have limited application to the specific challenges of aDNA in terms of degradation and chemical modifications that manifest as misincorporated nucleotides. Our analyses highlight the need for a pathogen identification pipeline that accommodates qualities of aDNA data and includes an essential and robust authentication for all ancient read assignments. HOPS provides a reliable and user-friendly solution to these established limitations.

HOPS was tested on simulated ancient pathogen DNA reads, and it detected all targeted species and successfully authenticated 90% of all cases in various metagenomic backgrounds with as few as 50 species-derived reads, representing less than 0.001% of the total dataset. In this context, our modified version of MALT, which tolerates mismatches resulting from DNA degradation, prevents a decrease in sensitivity even in cases of heavily damaged aDNA. For 500 reads, authentication was not possible for two *Streptococcus* and two *Neisseria* species in dental calculus. This is due to a strong background of similar species that is frequently found in this material. Oral streptoccoci were in fact identified in the calculus background by all programs. Thus, for these species, more reads are required for a successful authentication.

We demonstrate that the marker gene-based metagenomic profiling tools MIDAS and metaBIT have lower sensitivities for pathogen detection compared to HOPS, especially for low coverage data, which is typical of ancient DNA screening datasets. Although the sensitivity of Kraken was similar to HOPS, and while Kraken’s alignment-free k-mer matching is considerably faster than the precise alignments used in HOPS, Kraken is incapable of validating species assignment and aDNA authenticity, and thus has a lower specificity. This is most clearly demonstrated by our analysis of a metagenomic soil sample in which Kraken detected numerous false positives, including *Mycobacterium tuberculosis* and *Bordetella pertussis* (whooping cough). This is likely due to many soil-dwelling bacteria that harbor genetic similarities to these pathogens, such as diverse mycobacterial species and *Bordetella petrii*, a close relative to *B. pertussis* that is a common constituent of environmental datasets. These effects are further compounded by the fact that many environmental microbes have not been genomically characterized and are not part of any reference database, which only increases the potential of false assignments to well-sequenced pathogens. The alignment-based validation procedure implemented in HOPS minimizes such false positive assignments and thus offers greater accuracy in pathogen identification during screening when environmental backgrounds comprise the dominant molecular signal.

As a pipeline for the assessment of archaeogenetic data, metaBIT implements a variety of methods for the detailed assessment of metagenomic composition that also includes validation of aDNA damage patterns. metaBIT is based on MetaPhlAn2 [29], which employs a marker gene-based approach in the initial detection step similar to MIDAS. Pathogens in low abundance are thus frequently missed in its initial steps when applied to shallow sequencing data as demonstrated by our comparative benchmarking. SPARSE employs a hierarchically structured database and a probabilistic model in order to avoid false positive species detections. These features led to its high specificity in our test setting. For our simulated data, SPARSE is much more sensitive than MIDAS and metaBIT. However, when the number of pathogen reads is very low, the correct detection is frequently missed. In this context, HOPS can offer a higher sensitivity and can additionally provide details about all evaluated authenticity criteria. An integrated approach combining HOPS and SPARSE or metaBIT might be a promising future strategy for a detailed characterization of complete microbiomes while at the same time providing a high level of sensitivity for the detection and authentication of pathogen DNA. In particular, the analysis of ancient samples that preserve their original microbiome signature, such as dental calculus [47] or coprolites [48], would benefit from a combined application of methodologies by using SPARSE and/or metaBIT to assess the microbial make-up and HOPS for additional in-depth species authentication.

For all taxonomic classifiers, correct assignment of metagenomic reads is strongly dependent on the quality of the underlying reference sequences. Currently, we use a curated database for MALT that contains completed reference sequences and assemblies for bacteria from RefSeq (December 2016). Database sizes are constantly increasing, but much of this growth derives from the addition of redundant sequence data from model organisms, which also creates biases. In this context, SPARSE aims to mitigate the influence of database redundancy by hierarchically structuring reference sequences, which could be employed to further improve HOPS.

In addition, analysis of our simulated dataset allowed for evaluation of the taxonomic placement of each of the bacterial pathogens in our target list. It became apparent that for some targets the taxonomic species level is not sufficient for identification. This applies to historically important pathogens such as *Y. pestis* or *M. tuberculosis*. Here, evaluation of a higher taxonomic level such as “complex” is more reliable, while in the case of *Salmonella typhi* (typhoid fever) a lower level (subspecies) is favorable. Therefore, our simulations provide a valuable resource for optimization of pathogen screening approaches in general and a guideline to develop it for additional microbes.

Here, HOPS was evaluated for its success in screening for bacterial pathogens. Because the reference database is user defined and can be amended to include, for example, the NCBI full nucleotide collection [49] or hand-curated sets of reference genomes, tremendous flexibility exists in molecular detection, which could extend to viruses, fungi, and eukaryotic parasites.

## Conclusions

We present a reliable and user-friendly computational pathogen screening pipeline for ancient DNA that has the flexibility of handling large datasets. HOPS successfully identifies both simulated and actual ancient pathogen DNA within complex metagenomic datasets, exhibiting a higher sensitivity than MIDAS, metaBIT, or SPARSE and with fewer false positives than Kraken. HOPS provides a high level of automatization that allows for the screening of thousands of datasets with very little hands-on time, and it offers detailed visualizations and statistics at each evaluation step, enabling a high level of quality control and analytical transparency. HOPS is a powerful tool for high-throughput pathogen screening in large-scale archaeogenetic studies, producing reliable and reproducible results even from remains with exceptionally low levels of pathogen DNA. Such qualities make HOPS a valuable tool for pathogen detection in the rapidly growing field of archaeogenetics.

## Methods

### Implementation of MaltExtract

MaltExtract is implemented in Java. It integrates parts of MEGAN’s [39] source code for accessing the RMA file structure and functions from *forester* (https://github.com/cmzmasek/forester) for traversing the taxonomic tree.

### Simulating data to analyze read assignment using the MALT LCA algorithm

Depending on the database structure and sequence similarity between reference sequences, the naïve LCA [36] algorithm will assign reads to different taxonomic units. To inquire how reads are assigned to the taxonomic tree for 33 bacterial pathogens (Additional file 1: Table S2), we simulated ancient pathogen DNA reads using gargammel [50] and spiked them into five ancient metagenomic background datasets obtained from bone, dentine, dental calculus, and soil (Table 1). The simulated reads carry a unique identifier in their header in order to differentiate them from metagenomic background sequences, which exhibit either full damage patterns or attenuated damage patterns following UDG-half treatment [51]. To simulate aDNA damage in the pathogen sequences, we applied damage profiles obtained from previously published ancient *Yersinia pestis* genomes with [13] and without UDG-half [18] treatment. Simulated reads were processed with the NGS data processing pipeline EAGER [52] and spiked into the metagenomic backgrounds in different amounts (50, 500, or 5000 reads). For each metagenomic background, a typical screening sequencing depth of five million reads was used.

### Evaluation of the damage-tolerant version of MALT

To preserve damage patterns when mapping reads with MALT, we modified the source code and compared the performance of the modified and default versions.

We therefore created with gargammel [50] test samples that show twice the amount of damage (~ 40%) usually found in ancient samples [13]. Here, we compare both MALT versions for the bacterial pathogen *Yersinia pestis* (CO92 reference). Both versions of MALT were tested with 85%, 95%, and 99% minimum percent identity filtering, to investigate the effects of percent identity filtering on the read alignment of aDNA reads.

### Comparison of HOPS to Kraken, SPARSE, MIDAS, and metaBIT

HOPS was compared to four metagenomic taxonomic classification tools: Kraken (v 0.10.6) [31], SPARSE (v 2019-05-31) [46], MIDAS (v 1.3) [30], and metaBIT (v 1.0.1) [45]. We only executed the first step of MIDAS that matches reads to the marker gene database to determine species abundance. This step was executed on 24 cores with default parameters. The first step is sufficient, as any species undetected in this step would not be detected in the remaining ones. Kraken was set to use 32 cores to align the sample data against its reference database with the preload parameter to load the entire database into memory before starting k-mer alignment. In a second step, kraken-translate was executed to transform taxonomy IDs into proper species names.

For SPARSE, we reserved the default number of 20 cores, we used default parameters except for changing *minFreq* to 0.000001 and *minNum* to 5 which are the recommended settings for aDNA analysis.

SPARSE was only tested on the first replicate of the simulated data.

metaBIT was executed with default parameters and a total of 64 cores available.

For Kraken, metaBIT, MIDAS, and SPARSE, we judged a pathogen as correctly identified if at least one read matched to the correct species to account for the differences in the database contents, methodologies, and output formats.

For HOPS to judge a pathogen detected by MALT as authentic, it had to fulfill at least the first of the three hierarchical authenticity criteria, which is a declining edit distance distribution. HOPS version 1.0 and MaltExtract version 0.9 were used for this analysis.

### Databases

In our study, HOPS uses a database containing all complete prokaryotic reference genomes obtained from NCBI (December 1, 2016) with entries containing “multi” and “uncultured” removed (13 entries). In total, 6249 reference genomes are included in the database, including all major bacterial pathogens scrutinized here. For Kraken, we downloaded the bacterial database with Kraken’s kraken-build script (June 1, 2017). The Kraken database contains no strain references for *Clostridium sporogenes.* Otherwise, it contains at least one reference for all of the simulated bacterial pathogens (Additional file 1: Table S2). For MIDAS, we used the default reference database (May 24, 2016), which contained no representation of *Yersinia pseudotuberculosis*, *Bordetella pertussis*, and *Brucella melitensis*.

MIDAS was tested on all data with version 1.3 and the MIDAS database version 1.2.

metaBIT used the MetaPhlAn2 [29] database (version biobakery-metaphlan2-27f7e0c86785)

For SPARSE, we built a representative database by running

*sparse index --dbname refseq --update*

*sparse query --dbname refseq --default representative | sparse mapDB --dbname refseq --seqlist stdin --mapDB representative*

That resulted in a database containing bacteria and archea with an average nucleotide identity (ANI) of 98%.

### Positive controls

We compare the sensitivity and specificity of HOPS, MIDAS, SPARSE, metaBIT, and Kraken using 27 metagenomic datasets previously shown to be positive for one of four microbial pathogens: *Yersinia pestis, Mycobacterium tuberculosis*, *Salmonella enterica*, and *Helicobacter pylori* (Table 2). These positive control samples represent real metagenomic data and therefore contain an unknown number of modern species in addition to the actual recovered bacterial pathogen. Read counts across all samples ranged from 70,897 to 52,858,027 reads. While most datasets were generated by shotgun library screening, four datasets were enriched for pathogen DNA prior to sequencing using DNA capture methods. For all captured datasets and a subset of shotgun datasets, DNA was treated with UDG prior to library construction to remove DNA damage. Both types of datasets were included to evaluate the performance of HOPS on samples with different levels of DNA damage and pathogen abundance.

### Runtimes

To calculate the runtimes for HOPS, Kraken, SPARSE, metaBIT, and MIDAS, we used a subset of the simulated files. The subset consisted of all metagenomic background datasets spiked with 5000 reads without technical replicates resulting in a total of 330 metagenomic files. HOPS, Kraken, and metaBIT had 64 cores available, MIDAS 24, and SPARSE 20.

## Supplementary information

**Additional file 1.** Supplementary figures and tables.

**Additional file 2.** Review history.

## Data Availability

The complete source code of HOPS is available from GitHub under the GNU General Public License v3.0 (https://github.com/rhuebler/HOPS) [53]. HOPS (including MALT) is also available from Bioconda: https://bioconda.github.io/recipes/hops/README.html The source code versions used for the analyses in this manuscript have been archived on Zenodo: HOPS (pipeline controlling module) [54]: 10.5281/zenodo.3362248 MaltExtract [55]: 10.5281/zenodo.3362242 PostProcessing [56]: 10.5281/zenodo.3362316 For this study, HOPS uses a database containing all complete prokaryotic reference genomes obtained from NCBI (December 1, 2016) with entries containing “multi” and “uncultured” removed (13 entries). In total, 6249 reference genomes are included in the database, including all major bacterial pathogens scrutinized here. The HOPS database is available upon request.
