# Supplementary material for: HOPS: automated detection and authentication of pathogen DNA in archaeological remains
Source: Genome Biol. 2019 Dec 16;20:280. doi: 10.1186/s13059-019-1903-0 (PMC6913047; doi:10.1186/s13059-019-1903-0)
Supplement: Supplementary file 1 — Additional file 1. Supplementary figures and tables. [file 13059_2019_1903_MOESM1_ESM.docx]

**Supplementary Information**

**HOPS: Automated detection and authentication of pathogen DNA in archaeological remains**

**Figure S1** The first and third steps in the HOPS postprocessing protocol require a decline in the Edit Distance distribution. The formula divides the absolute sum of all negative neighboring differences (ie. declining) by the absolute sum of all differences (including positive).

**Figure S2** HOPS summary output for a tuberculosis positive sample. Upper left: Edit distance distribution for all reads assigned to M. tuberculosis. Upper right: Edit distance distribution for assigned reads that show a possible DNA damage signal. Middle left: DNA damage plot for assigned reads. Lower left: Top ten references with percentage of aligned reads. Middle right: Summary statistics for assigned reads.

**Table S1** Results for negative controls. For HOPS the step in the post processing that was reached for each species is indicated (0: No detection; 1: detected with declining edit distance distribution; 2: additionally indication for damage; 3: additionally declining edit distance distribution for damaged reads). For Kraken the number of k-mers assigned to the species and for MIDAS the number of assigned reads is listed.

| Species | bone_UDGhalf_5m_TOSM1a | calculus_UDGno_5m_A081 | dentine_UDGhalf_5m_MK5 | dentine_UDGno_5m_LP39.10_pB_l5 | soil_UDGno_5m_tepos | Classifier |
| --- | --- | --- | --- | --- | --- | --- |
| Clostridium_botulinum | 0 | 0 | 0 | 0 | 2 | HOPS |
| Clostridium_tetani | 0 | 0 | 0 | 0 | 1 | HOPS |
| Clostridium_tetani_E88 | 0 | 0 | 0 | 0 | 0 | HOPS |
| Neisseria meningitidis | 0 | 0 | 2 | 0 | 0 | HOPS |
| Porphyromonas_gingivalis | 0 | 3 | 0 | 0 | 0 | HOPS |
| Porphyromonas_gingivalis_W83 | 0 | 2 | 0 | 0 | 0 | HOPS |
| Streptococcus_gordonii_str._Challis_substr._CH1 | 0 | 0 | 0 | 1 | 0 | HOPS |
| Streptococcus_mutans | 0 | 3 | 0 | 2 | 0 | HOPS |
| Tannerella_forsythia | 0 | 0 | 0 | 0 | 0 | HOPS |
| Treponema_denticola_ATCC_35405 | 0 | 2 | 0 | 1 | 0 | HOPS |
|  | bone_UDGhalf_5m_TOSM1a | calculus_UDGno_5m_A081 | dentine_UDGhalf_5m_MK5 | dentine_UDGno_5m_LP39.10_pB_l5 | soil_UDGno_5m_tepos | Kraken |
| Bacillus anthracis | 0 | 5 | 1 | 0 | 0 | Kraken |
| Bordetella pertussis | 1 | 12 | 20 | 4 | 10 | Kraken |
| Borrelia burgdorferi B31 | 0 | 0 | 0 | 0 | 0 | Kraken |
| Brucella abortus | 0 | 0 | 0 | 0 | 1 | Kraken |
| Brucella melitensis | 0 | 4 | 0 | 0 | 1 | Kraken |
| Clostridium botulinum | 40 | 182 | 0 | 21 | 40 | Kraken |
| Clostridium sporogenes | 0 | 0 | 0 | 0 | 0 | Kraken |
| Clostridium tetani | 13 | 50 | 1 | 2 | 240 | Kraken |
| Corynebacterium diphtheriae | 4 | 79 | 9 | 13 | 11 | Kraken |
| Haemophilus influenzae | 2 | 1617 | 0 | 2 | 3 | Kraken |
| Helicobacter pylori | 8 | 12 | 0 | 0 | 4 | Kraken |
| Mycobacterium avium subsp. paratuberculosis | 13 | 8 | 39 | 6 | 26 | Kraken |
| Mycobacterium leprae | 16 | 19 | 22 | 13 | 21 | Kraken |
| Mycobacterium tuberculosis | 53 | 46 | 208 | 55 | 117 | Kraken |
| Neisseria gonorrhoeae | 4 | 2407 | 2 | 3 | 8 | Kraken |
| Neisseria meningitidis | 4 | 4448 | 0 | 9 | 12 | Kraken |
| Porphyromonas gingivalis | 2 | 13925 | 0 | 6 | 0 | Kraken |
| Salmonella enterica subsp. enterica | 8 | 54 | 16 | 24 | 16 | Kraken |
| Staphylococcus aureus subsp. aureus | 1 | 19 | 2 | 4 | 4 | Kraken |
| Streptococcus gordonii | 0 | 71751 | 0 | 15 | 0 | Kraken |
| Streptococcus mutans | 0 | 401 | 0 | 17 | 0 | Kraken |
| Streptococcus pneumoniae | 0 | 3224 | 0 | 0 | 0 | Kraken |
| Tannerella forsythia | 8 | 31294 | 14 | 18 | 14 | Kraken |
| Treponema denticola | 0 | 25264 | 0 | 4 | 0 | Kraken |
| Treponema pallidum subsp. pallidum | 0 | 0 | 0 | 0 | 0 | Kraken |
| Vibrio cholerae | 6 | 60 | 1 | 6 | 5 | Kraken |
| Yersinia pestis | 0 | 4 | 0 | 0 | 0 | Kraken |
| Yersinia pseudotuberculosis | 15 | 21 | 7 | 39 | 7 | Kraken |
|  | bone_UDGhalf_5m_TOSM1a | calculus_UDGno_5m_A081 | dentine_UDGhalf_5m_MK5.001 | dentine_UDGno_5m_LP39.10_pB_l5 | soil_UDGno_5m_tepos | metaBIT |
| Bacillus anthracis | 0 | 0 | 0 | 0 | 0 | metaBIT |
| Bordetella pertussis | 0 | 0 | 0 | 0 | 0 | metaBIT |
| Borrelia burgdorferi B31 | 0 | 0 | 0 | 0 | 0 | metaBIT |
| Brucella abortus | 0 | 0 | 0 | 0 | 0 | metaBIT |
| Brucella melitensis | 0 | 0 | 0 | 0 | 0 | metaBIT |
| Clostridium botulinum | 0 | 0 | 0 | 0 | 0 | metaBIT |
| Clostridium sporogenes | 0 | 0 | 0 | 0 | 0 | metaBIT |
| Clostridium tetani | 0 | 0 | 0 | 0 | 0 | metaBIT |
| Corynebacterium diphtheriae | 0 | 0 | 0 | 0 | 0 | metaBIT |
| Haemophilus influenzae | 0 | 0 | 0 | 0 | 0 | metaBIT |
| Helicobacter pylori | 0 | 0 | 0 | 0 | 0 | metaBIT |
| Mycobacterium avium | 0 | 0 | 0 | 0 | 0 | metaBIT |
| Mycobacterium leprae | 0 | 0 | 0 | 0 | 0 | metaBIT |
| Mycobacterium tuberculosis | 0 | 0 | 0 | 0 | 0 | metaBIT |
| Neisseria gonorrhoeae | 0 | 0 | 0 | 0 | 0 | metaBIT |
| Neisseria meningitidis | 0 | 0 | 0 | 0 | 0 | metaBIT |
| Porphyromonas gingivalis | 0 | 531 | 0 | 0 | 0 | metaBIT |
| Salmonella enterica | 0 | 0 | 0 | 0 | 0 | metaBIT |
| Staphylococcus aureus | 0 | 0 | 0 | 0 | 0 | metaBIT |
| Streptococcus gordonii | 0 | 570 | 0 | 0 | 0 | metaBIT |
| Streptococcus mutans | 0 | 0 | 0 | 0 | 0 | metaBIT |
| Streptococcus pneumoniae | 0 | 0 | 0 | 0 | 0 | metaBIT |
| Tannerella forsythia | 0 | 794 | 0 | 0 | 0 | metaBIT |
| Treponema denticola | 0 | 912 | 0 | 0 | 0 | metaBIT |
| Treponema pallidum | 0 | 0 | 0 | 0 | 0 | metaBIT |
| Vibrio cholerae | 0 | 0 | 0 | 0 | 0 | metaBIT |
| Yersinia pestis | 0 | 0 | 0 | 0 | 0 | metaBIT |
| Yersinia pseudotuberculosis | 0 | 0 | 0 | 0 | 0 | metaBIT |
|  | bone_UDGhalf_5m_TOSM1a | calculus_UDGno_5m_A081 | dentine_UDGhalf_5m_MK5 | dentine_UDGno_5m_LP39.10_pB_l5 | soil_UDGno_5m_tepos | MIDAS |
| Bacillus anthracis | 0 | 0 | 0 | 0 | 0 | MIDAS |
| Bordetella pertussis | 0 | 0 | 0 | 0 | 0 | MIDAS |
| Borrelia burgdorferi B31 | 0 | 0 | 0 | 0 | 0 | MIDAS |
| Brucella abortus | 0 | 0 | 0 | 0 | 0 | MIDAS |
| Brucella melitensis | 0 | 0 | 0 | 0 | 0 | MIDAS |
| Clostridium botulinum | 0 | 0 | 0 | 0 | 0 | MIDAS |
| Clostridium sporogenes | 0 | 0 | 0 | 0 | 0 | MIDAS |
| Clostridium tetani | 0 | 0 | 0 | 0 | 0 | MIDAS |
| Corynebacterium diphtheriae | 0 | 0 | 0 | 0 | 0 | MIDAS |
| Haemophilus influenzae | 0 | 0 | 0 | 0 | 0 | MIDAS |
| Helicobacter pylori | 0 | 0 | 0 | 0 | 0 | MIDAS |
| Mycobacterium avium | 0 | 0 | 0 | 0 | 0 | MIDAS |
| Mycobacterium leprae | 0 | 0 | 0 | 0 | 0 | MIDAS |
| Mycobacterium tuberculosis | 0 | 0 | 0 | 0 | 0 | MIDAS |
| Neisseria gonorrhoeae | 0 | 0 | 0 | 0 | 0 | MIDAS |
| Neisseria meningitidis | 0 | 0 | 0 | 0 | 0 | MIDAS |
| Porphyromonas gingivalis | 0 | 29 | 0 | 0 | 0 | MIDAS |
| Salmonella enterica | 0 | 0 | 0 | 0 | 0 | MIDAS |
| Staphylococcus aureus | 0 | 0 | 0 | 0 | 0 | MIDAS |
| Streptococcus gordonii | 0 | 62 | 0 | 0 | 0 | MIDAS |
| Streptococcus mutans | 0 | 0 | 0 | 0 | 0 | MIDAS |
| Streptococcus pneumoniae | 0 | 1 | 0 | 0 | 0 | MIDAS |
| Tannerella forsythia | 0 | 26 | 0 | 0 | 0 | MIDAS |
| Treponema denticola | 0 | 42 | 0 | 0 | 0 | MIDAS |
| Treponema pallidum | 0 | 0 | 0 | 0 | 0 | MIDAS |
| Vibrio cholerae | 0 | 0 | 0 | 0 | 0 | MIDAS |
| Yersinia pestis | 0 | 0 | 0 | 0 | 0 | MIDAS |
| Yersinia pseudotuberculosis | 0 | 0 | 0 | 0 | 0 | MIDAS |
|  | bone_UDGhalf_5m_TOSM1a | calculus_UDGno_5m_A081 | dentine_UDGhalf_5m_MK5 | dentine_UDGno_5m_LP39.10_pB_l5 | soil_UDGno_5m_tepos | SPARSE |
| Bacillus anthracis | 0 | 0 | 0 | 0 | 0 | SPARSE |
| Bordetella pertussis | 0 | 0 | 0 | 0 | 0 | SPARSE |
| Borrelia burgdorferi B31 | 0 | 0 | 0 | 0 | 0 | SPARSE |
| Brucella abortus | 0 | 0 | 0 | 0 | 0 | SPARSE |
| Brucella melitensis | 0 | 0 | 0 | 0 | 0 | SPARSE |
| Clostridium botulinum | 0 | 0 | 0 | 85 | 0 | SPARSE |
| Clostridium sporogenes | 0 | 0 | 0 | 0 | 0 | SPARSE |
| Clostridium tetani | 0 | 0 | 0 | 0 | 117 | SPARSE |
| Corynebacterium diphtheriae | 0 | 0 | 0 | 0 | 0 | SPARSE |
| Haemophilus influenzae | 0 | 403 | 0 | 0 | 0 | SPARSE |
| Helicobacter pylori | 0 | 0 | 0 | 0 | 0 | SPARSE |
| Mycobacterium avium | 0 | 0 | 0 | 0 | 0 | SPARSE |
| Mycobacterium leprae | 0 | 0 | 0 | 0 | 0 | SPARSE |
| Mycobacterium tuberculosis | 0 | 0 | 0 | 0 | 0 | SPARSE |
| Neisseria gonorrhoeae | 0 | 0 | 0 | 0 | 0 | SPARSE |
| Neisseria meningitidis | 0 | 222 | 0 | 0 | 0 | SPARSE |
| Porphyromonas gingivalis | 0 | 6431 | 0 | 0 | 0 | SPARSE |
| Salmonella enterica | 0 | 0 | 0 | 0 | 0 | SPARSE |
| Staphylococcus aureus | 0 | 0 | 0 | 0 | 0 | SPARSE |
| Streptococcus gordonii | 0 | 15266 | 0 | 0 | 0 | SPARSE |
| Streptococcus mutans | 0 | 93 | 0 | 0 | 0 | SPARSE |
| Streptococcus pneumoniae | 0 | 20 | 0 | 0 | 0 | SPARSE |
| Tannerella forsythia | 0 | 8975 | 0 | 0 | 0 | SPARSE |
| Treponema denticola | 0 | 11575 | 0 | 0 | 0 | SPARSE |
| Treponema pallidum | 0 | 0 | 0 | 0 | 0 | SPARSE |
| Vibrio cholerae | 0 | 0 | 0 | 0 | 0 | SPARSE |
| Yersinia pestis | 0 | 0 | 0 | 0 | 0 | SPARSE |
| Yersinia pseudotuberculosis | 0 | 0 | 0 | 0 | 0 | SPARSE |

**Table S2** Genomes used to generate simulated ancient pathogen DNA data sets

| **Bacillus anthracis str Ames** | **Neisseria meningitidis MC58** |
| --- | --- |
| **Bordetella pertussis Tohama I** | **Porphyromonas gingivalis W83** |
| **Borrelia burgdorferi B31** | **Salmonella enterica subsp enterica serovar Enteritidis str P125109** |
| **Brucella abortus 2308** | **Salmonella enterica subsp enterica serovar Typhi str CT18** |
| **Brucella melitensis bv 1 str 16M** | **Salmonella enterica subsp enterica serovar Typhimurium str LT2** |
| **Clostridium botulinum A str ATCC 3502** | **Staphylococcus aureus subsp aureus NCTC 8325** |
| **Clostridium botulinum BKT015925** | **Streptococcus gordonii str Challis substr CH1** |
| **Clostridium botulinum E3 str Alaska E43** | **Streptococcus mutans UA159** |
| **Clostridium sporogenes NCIMB 10696** | **Streptococcus pneumoniae R6** |
| **Clostridium tetani E88** | **Tannerella forsythia 92A2** |
| **Corynebacterium diphtheriae NCTC 13129** | **Treponema denticola ATCC 35405** |
| **Haemophilus influenzae Rd KW20** | **Treponema pallidum subsp pallidum str nichols** |
| **Helicobacter pylori 26695** | **Vibrio cholerae M66-2** |
| **Mycobacterium avium subsp paratuberculosis K10** | **Vibrio cholerae O1 biovar El Tor str N16961** |
| **Mycobacterium leprae TN** | **Yersinia pestis CO92** |
| **Mycobacterium tuberculosis anc** | **Yersinia pseudotuberculosis IP31758** |
| **Neisseria gonorrhoeae FA 1090** |  |
