# Supplementary material for: HOPS: automated detection and authentication of pathogen DNA in archaeological remains
Source: Genome Biol. 2019 Dec 16;20:280. doi: 10.1186/s13059-019-1903-0 (PMC6913047; doi:10.1186/s13059-019-1903-0)
Supplement: Supplementary file 2 — Additional file 2. Review history. [file 13059_2019_1903_MOESM2_ESM.docx]

Review History

**First round of review**

**Reviewer 1**

**Were you able to assess all statistics in the manuscript, including the appropriateness of statistical tests used?**

There are no statistics in the manuscript.

**Were you able to directly test the methods?**

No

**Comments to author:**

Please consider the following questions:

-Is the Software likely to be of broad utility? Is it easy to install and use?

Installing the software is not easy for non Java users. As this is of general interest to a wider audience. Do not assume the reads are familiar with Java.

-Does the software represent a significant advance over previously published tools (as demonstrated by direct comparison with available related software)?

HOPS is an add on to MALT (the MEGAN aligner) that extracts value out of the alignments generated by MALT. These alignments allow inferring species information according to the authors.

-Please indicate briefly the novel features and/or advantages of the software and/or please reference the relevant publications and which alternatives, if any, it should be compared with.

See above. I am not aware of a similar tools.

-Are the methods appropriate to the aims of the study, are they well described, and are necessary controls included?

The authors tested on real and simulated data. This is the best they can.

-Are the conclusions adequately supported by the data shown?

The authors claim to have produced a fast tool, being slower than Kraken (and likely slower than most tools on the market) this reviewer would suggest removing the word fast. They trade run time for accuracy, which is acceptable. However the computational cost for HOPS is high.

-Are sufficient details provided to allow replication and comparison with related analyses that may have been performed?

NO. The authors should consider a Docker container with the software pre-installed and or a CWL tool description to allow third parties to use their tool without having to learn modern Java.

-Is the paper of broad interest to others in the field, or of outstanding interest to a broad audience of biologists?

This paper is relevant for a small section of the field, within that fields it has merits.

**Reviewer 2**

**Were you able to assess all statistics in the manuscript, including the appropriateness of statistical tests used?**

There are no statistics in the manuscript.

**Were you able to directly test the methods?**

No

**Comments to author:**

The HOPS software does indeed seem to have important utility for the aDNA utility. My major concerns are with installing the software. The process seems complicated and non-intuitive. For example, when I click on maltextract, the readme wants to redirect back to HOPS and doesn't include any information on installation. For broad utility, HOPS should be packaged in conda or docker. Some specific comments/questions:

L69: You might think of mentioning that bacteria typically have multiple copies of rRNA

L164: What is meant by 'edit distance'. An explicit definition would be helpful.

L351: It would be nice to also list the memory requirements required and if processes could be performed in parallel

L410: Why wasn't metaBIT a part of your computational comparisons? This seems like it would be the most apples-to-apples type of comparison.

L470: Is "complex" a modifier in the MALT database? It would likely not be a qualifier in NT, correct?

L479: It is unclear exactly what "EAGER" is doing. Please expand

The MALT database seems quite out of date. Would be nice to discuss how using NT, with its potential taxonomic problems, could affect the analyses

**Reviewer 3**

**Were you able to assess all statistics in the manuscript, including the appropriateness of statistical tests used?**

No, I do not feel adequately qualified to assess the statistics.

**Were you able to directly test the methods?**

No

**Comments to author:**

Ron Hübler and collaborators report on a bioinformatic pipeline for retrieving microbial sequences in «ancient» metagenomes. In this report it is unclear whether HOPS retrieves any bacterium, or is limited to the list of 33 "pathogens" illustrating this report. This point may be clarified by retrieving all the bacterial reads in the tested datasets. The authors need to clarify whether HOPS was designed to retrieve one particular "pathogen" (in fact, DNA organism) in datasets, or blindly explore the list of DNA-organisms in the datasets. At last, only blind testing of datasets is of value, testing "spiked" datasets is poorly informative.

Accordingly, this contributive report could be strengthened by consideration of the following minor remarks:

1. Line 41: Coprolites and dental pulp may be added to the list

2. Line 73: In fact, 16S is not specific.

3. Line 87: The authors may want to document this affirmation regarding microbial DNA (not eukaryotic DNA) by citing reference to microbes

4. Line 96: and text in general: The authors may want to replace "pathogen" by "microbe" or something as "microbes including pathogens" (or bacteria, as HOPS seem to address only sequences of supposed bacterial origin). Dealing with very ancient metagenomes, it may be too presumptive to classify microbes as pathogens (implicating the others being non-pathogens).

5. Lines 199-204: See above remark n°3. Please document the de-amination phenomenon, on bacterial aDNA which is the topic of present study, not human (eukaryotic) aDNA which is not the topic.

- Lines 242-244: Unclear, where is the HOPS' mistake?

- Line 312: It should be useful by this "negative" control section, before presenting "spiked" metagenomes, to a more accurate interpretation of "spiked" data.

- Line 520: Helicobacter

- Lines 346-347: Authors should list the 5 missed "pathogens", data are not presented.

- Is there any difference between the various ancient specimens?

- Lines 346-371: These vague, poorly contributive sentences could be deleted; or referenced with references dealing with bacterial aDNA.

- Lines 373-374: It is unclear in this report if HOPS performing bacterial aDNA sequence retrieving, or retrieving sequences from a set list of bacteria ("pathogen"). If HOPS performing bacterial aDNA retrieving, why not viral/eukaryal aDNA retrieving. Unclear.

**Authors Response**

**Point-by-point responses to the reviewers’ comments:**

Reviewer #1:

Please consider the following questions:

-Is the Software likely to be of broad utility? Is it easy to install and use?

Installing the software is not easy for non Java users. As this is of general interest to a wider audience. Do not assume the reads are familiar with Java.

*Answer: We now provide a conda package for the installation of HOPS, which sets up HOPS, MALT and all required dependencies. Using conda packages is well established in the bioinformatic community and a convenient solution also for users who are not familiar with Java.*

The authors claim to have produced a fast tool, being slower than Kraken (and likely slower than most tools on the market) this reviewer would suggest removing the word fast. They trade run time for accuracy, which is acceptable. However the computational cost for HOPS is high.

*Answer: It is correct that HOPS has a higher runtime in comparison to other tools, that do not calculate alignments to whole genomes. We have removed the term ‚fast’ from the description.*

-Are sufficient details provided to allow replication and comparison with related analyses that may have been performed?

NO. The authors should consider a Docker container with the software pre-installed and or a CWL tool description to allow third parties to use their tool without having to learn modern Java.

*Answer: Agreed. See above. We now provide a conda package for the installation of HOPS.*

Reviewer #2: ===

The HOPS software does indeed seem to have important utility for the aDNA utility. My major concerns are with installing the software. The process seems complicated and non-intuitive. For example, when I click on maltextract, the readme wants to redirect back to HOPS and doesn't include any information on installation. For broad utility, HOPS should be packaged in conda or docker. Some specific comments/questions:

*Answer: MaltExtract now has an independent readme and github release so users only interested in the MaltExtract portion of HOPS can download a prebuilt jar file and do not have to engage with the rest of HOPS. Regarding the installation of HOPS we now provide a conda package as suggested by the reviewer.*

L69: You might think of mentioning that bacteria typically have multiple copies of rRNA

*Answer: We added this information to the manuscript (line 72).*

L164: What is meant by 'edit distance'. An explicit definition would be helpful.

*Answer: We have added a description of the term edit distance to the manuscript (line 93).*

L351: It would be nice to also list the memory requirements required and if processes could be performed in parallel

*Answer: We added this information to the manuscript (Runtime section).*

L410: Why wasn't metaBIT a part of your computational comparisons? This seems like it would be the most apples-to-apples type of comparison.

*Answer: We have now added metaBIT as well as SPARSE to our comparison. We can show that HOPS outperforms both tools at low pathogen DNA concentrations.*

L470: Is "complex" a modifier in the MALT database? It would likely not be a qualifier in NT, correct?

*Answer: The modifier „complex“ is defined as part of the NCBI taxonomy and is part of the MALT database as well as NT.*

L479: It is unclear exactly what "EAGER" is doing. Please expand

*Answer: EAGER is an NGS data processing pipeline. We have added this information to the manuscript (line 535).*

The MALT database seems quite out of date. Would be nice to discuss how using NT, with its potential taxonomic problems, could affect the analyses

*Answer: The focus of our manuscript is the detection of ancient DNA from major human and animal bacterial pathogens. All those are well represented in our database, which was selected for that purpose in December 2016. The reviewer is correct that larger databases are available (eg. NT), which however need to be carefully examined in order to avoid spurious results (or lack of results) due to taxonomical mislabels and subsequent misalignments. We have added that notion to the Discussion and forward the reader to two recent reviews that discuss the aforementioned challenges in great detail.*

Reviewer #3: Ron Hübler and collaborators report on a bioinformatic pipeline for retrieving microbial sequences in «ancient» metagenomes. In this report it is unclear whether HOPS retrieves any bacterium, or is limited to the list of 33 "pathogens" illustrating this report. This point may be clarified by retrieving all the bacterial reads in the tested datasets. The authors need to clarify whether HOPS was designed to retrieve one particular "pathogen" (in fact, DNA organism) in datasets, or blindly explore the list of DNA-organisms in the datasets. At last, only blind testing of datasets is of value, testing "spiked" datasets is poorly informative.

*Answer: We agree with the reviewer that only a tool that blindly tests is of value. HOPS blindly tests (ancient) metagenomic datasets for any user-defined set of taxonomic groups. We have made this point clearer in the manuscript. We evaluate and compare HOPS using published ancient metagenomics datasets as well as simulated reads spiked into authentic ancient metagenomic control datasets. We show that HOPS identifies and authenticates successfully all bacterial pathogens of interest.*

Accordingly, this contributive report could be strengthened by consideration of the following minor remarks:

1. Line 41: Coprolites and dental pulp may be added to the list

*Answer: Here we focus on sources for ancient metagenomic data that have been used in the past to describe ancient bacterial pathogens by mostly targeted bacterial identification, incl. dental pulp. However, we agree that in the future HOPS can be used also for the detection of ancient bacteria in other sources such as coprolites even though we have not evaluated HOPS using coprolite data.*

2. Line 73: In fact, 16S is not specific.

*Answer: We have changed our language in the manuscript accordingly (line 75).*

3. Line 87: The authors may want to document this affirmation regarding microbial DNA (not eukaryotic DNA) by citing reference to microbes

*Answer: We have added references accordingly (line 91).*

4. Line 96: and text in general: The authors may want to replace "pathogen" by "microbe" or something as "microbes including pathogens" (or bacteria, as HOPS seem to address only sequences of supposed bacterial origin). Dealing with very ancient metagenomes, it may be too presumptive to classify microbes as pathogens (implicating the others being non-pathogens).

*Answer: HOPS is designed with the idea to discover ancient bacterial pathogens, which we try to convey by using the indeed somewhat unspecific term pathogen. We have now made this nomenclature clearer to the reader in the abstract, introduction and throughout the text.*

5. Lines 199-204: See above remark n°3. Please document the de-amination phenomenon, on bacterial aDNA which is the topic of present study, not human (eukaryotic) aDNA which is not the topic.

*Answer: We have added references accordingly (line 205).*

- Lines 242-244: Unclear, where is the HOPS' mistake?

*Answer: The species assignment is still correct. However, the strain is a different one than originally simulated. We have clarified this in the text (lines 249-251).*

- Line 312: It should be useful by this "negative" control section, before presenting "spiked" metagenomes, to a more accurate interpretation of "spiked" data.

*Answer: We consider the order as is more intuitive for the reader and report assessment of sensitivity first, followed by an assessment of specificity.*

- Line 520: Helicobacter

*Answer: Corrected.*

- Lines 346-347: Authors should list the 5 missed "pathogens", data are not presented.

*Answer: We have added this information as part of Table 2.*

- Is there any difference between the various ancient specimens?

*Answer: Most importantly from the perspective of pathogen detection there are differences in the number of sequenced reads, library processing (capture vs. shotgun) and the bacterial pathogen species the samples are positive for. These differences are listed in Table 2.*

- Lines 346-371: These vague, poorly contributive sentences could be deleted; or referenced with references dealing with bacterial aDNA.

*Answer: We have added references accordingly (line 413).*

- Lines 373-374: It is unclear in this report if HOPS performing bacterial aDNA sequence retrieving, or retrieving sequences from a set list of bacteria ("pathogen"). If HOPS performing bacterial aDNA retrieving, why not viral/eukaryal aDNA retrieving. Unclear.

*Answer: HOPS is able to retrieve and assess sequence alignment information for any taxonomic node in the database. However, HOPS provides a standard list of taxa in the github repository which can be altered/extended by the user. Here, we focused on evaluating HOPS on bacterial species, but viral and eukaryotic organisms can be included as well, which we acknowledge in the discussion (lines 498-502).*

**Second round of review**

**Reviewer 2**

Overall, the authors did a good job of addressing my questions.

I installed the code with bioconda, but can't test the code without a config file. On the github page, the authors claim that there is an example config file at: "/projects1/clusterhomes/huebler/RMASifter/amps/". This looks to be a private server. The authors should provide this file as part of the github repo. It would be great if the authors also posted reads that could be used for testing/validation.
